# X-ray fan beam coded aperture transmission and diffraction imaging for fast material analysis

**DOI:** 10.1038/s41598-021-90163-0

**Published:** 2021-05-19

**Authors:** Stefan Stryker, Joel A. Greenberg, Shannon J. McCall, Anuj J. Kapadia

**Affiliations:** 1 Medical Physics Graduate Program, Duke University, Durham, NC USA; 2 Department of Electrical and Computer Engineering, Duke University, Durham, NC USA; 3 Department of Pathology, Duke University Medical Center, Durham, NC USA; 4 Carl E. Ravin Advanced Imaging Laboratories, Department of Radiology, Duke University, DUMC Box 2731, Durham, NC 27708 USA

**Keywords:** Imaging techniques, Cancer imaging

## Abstract

X-ray transmission imaging has been used in a variety of applications for high-resolution measurements based on shape and density. Similarly, X-ray diffraction (XRD) imaging has been used widely for molecular structure-based identification of materials. Combining these X-ray methods has the potential to provide high-resolution material identification, exceeding the capabilities of either modality alone. However, XRD imaging methods have been limited in application by their long measurement times and poor spatial resolution, which has generally precluded combined, rapid measurements of X-ray transmission and diffraction. In this work, we present a novel X-ray fan beam coded aperture transmission and diffraction imaging system, developed using commercially available components, for rapid and accurate non-destructive imaging of industrial and biomedical specimens. The imaging system uses a 160 kV Bremsstrahlung X-ray source while achieving a spatial resolution of ≈ 1 × 1 mm^2^ and a spectral accuracy of > 95% with only 15 s exposures per 150 mm fan beam slice. Applications of this technology are reported in geological imaging, pharmaceutical inspection, and medical diagnosis. The performance of the imaging system indicates improved material differentiation relative to transmission imaging alone at scan times suitable for a variety of industrial and biomedical applications.

## Introduction

X-ray technology has been utilized within imaging applications^1^ since the 1890s. The phenomenon commonly exploited within transmission X-ray imaging is the varying extent to which materials absorb X-rays, which gives rise to structure and attenuation information^2,3^. Given the non-uniqueness of some objects’ overall shapes and densities, transmitted X-rays are often not sufficient to uniquely identify or differentiate these materials from one another. In contrast to transmission imaging, X-ray diffraction (XRD) measures the intensity distribution in energy and/or angle of X-rays that directly interact with a material’s molecular structure. The molecular structure of a material results in unique constructive and destructive interference of scattered X-rays^4^, giving rise to unique XRD spectra. As a result, XRD has the capability of identifying specific materials (including those in non-crystalline form) with high accuracy.

While non-imaging-based XRD is utilized widely in applications such as extra-terrestrial sample analysis^5^, drug inspection^6–8^, and material science^9,10^, challenges arising from implementing measurements at relevant spatial resolutions and scan times prevent XRD from being widely used in imaging applications. Commercial diffractometers, the most widely used XRD architecture, utilize X-ray pencil beam geometries operating at low X-ray energies (e.g., the most commonly used copper anodes have a k-alpha energy of 8.05 keV), which allows for highly accurate localized identification of scatter on the surface of materials. While these systems can be used to generate planar XRD images of objects via raster scanning in two dimensions^11,12^, this process requires significant sample preparation and is typically slow (often requiring hours to measure small objects^13,14^). Furthermore, the low penetration depth of the X-rays used in diffractometers limits the XRD analysis to only a thin surface layer of the object. On the other hand, synchrotron-based XRD imaging^15^ requires highly advanced and specialized facilities that are not accessible to all scientists or clinicians. XRD imaging, in shorter scan times and smaller laboratory and clinical environments, remains highly desirable and has been the subject of recent exploration^16,17^.

**Figure 1:**
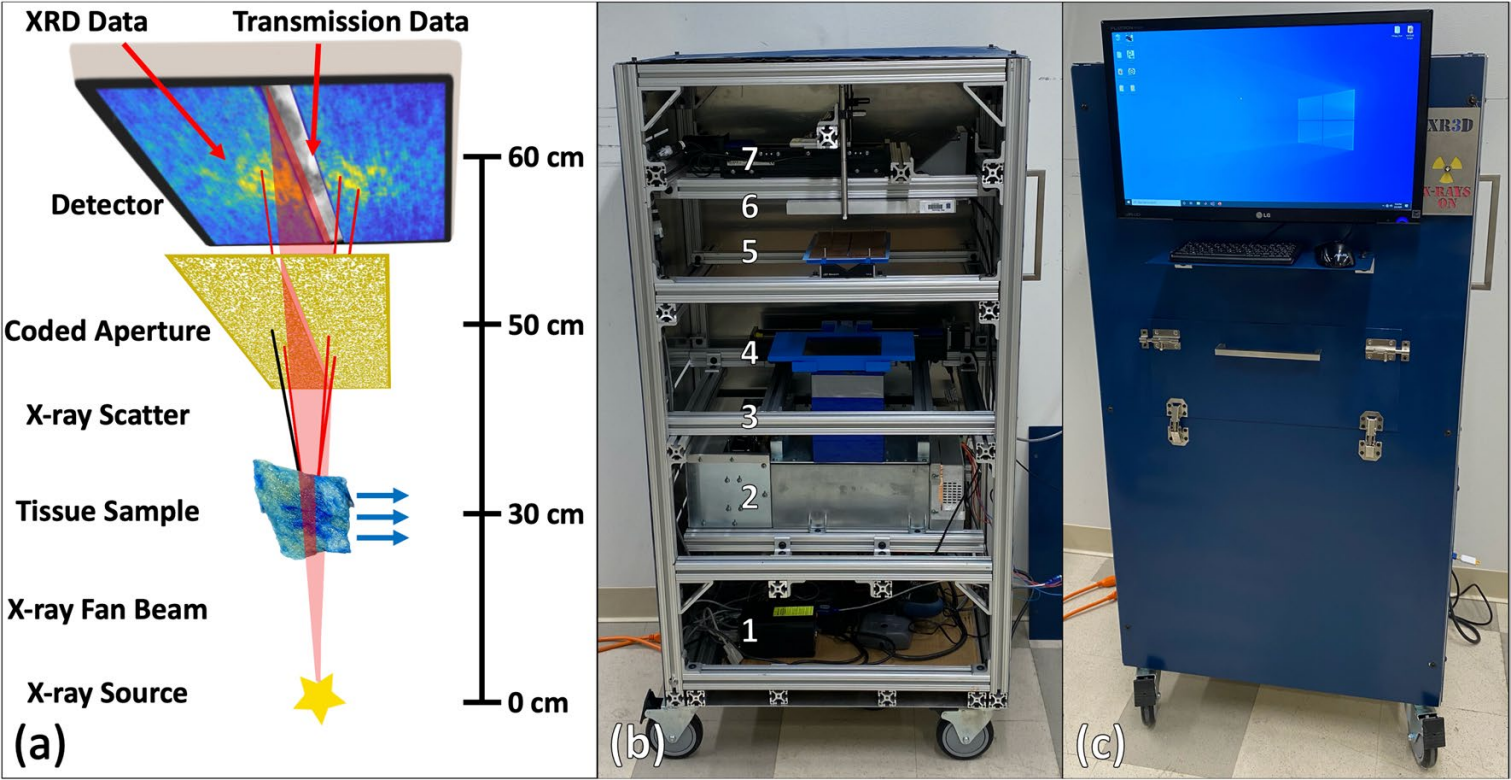
The X-ray fan beam coded aperture scanner developed in this work. (**a**) Schematic representation of the scanner, with blue arrows representing scanned materials being translated through the X-ray fan beam to collect transmission and XRD data for the entire sample. Images on the detector represent the captured transmission data (greyscale) and multiplexed XRD data (blue-yellow) that are captured sequentially. (**b**) Internal components of scanner with labels corresponding to (1) computer/controllers, (2) X-ray generator, (3) collimation to produce fan beam, (4) motorized tray for translating materials through fan beam, (5) coded aperture with central slit for fan beam, (6) X-ray detector and (7) motorized lead block for shielding fan beam during XRD data acquisition. (**c**) Finished system with internal lead shielding, monitor, and access panel for placing materials inside the scanner. Procreate (v5) was used in the graphical design of (**a**).

In this work, we present an X-ray fan beam coded aperture transmission and diffraction imaging system for rapid and accurate non-destructive imaging of industrial and biomedical specimens. We demonstrate the ability to accurately measure XRD spectra throughout a sample’s extent, providing molecular structure-based information at speeds relevant to industrial or clinical settings. Consistent with our previous simulations^18^, we demonstrate that the X-ray fan beam coded aperture approach increases the throughput of XRD imaging (i.e. reducing the scan time) and requires raster scanning in only one dimension while maintaining reasonably high spatial resolution^19^. Our system was designed and built using predominantly off-the-shelf components in a compact footprint that enables mobility. The system is capable of achieving ≈ 1 × 1 mm^2^ XRD spatial resolution^18^ with 95% accuracy of XRD spectral shape (using a standard Bremsstrahlung X-ray source—spectrum shown in supplemental Fig. S1), while operating at scan speeds of 15 s/150 mm fan slice. A schematic of the system, the internal placement of key components, and the finished scanner are shown in Fig. 1. As an initial exploration of applications, data were collected from geological, pharmaceutical, and medical specimens to evaluate and demonstrate the system’s utility in combined high-resolution imaging and material identification.

## Results

### Initial characterization of X-ray fan beam coded aperture imaging system

The X-ray fan beam coded aperture approach makes parallelized, multiplexed measurements to increase throughput/signal while relying on computational post-processing to recover spatially-resolved information^20^. As shown in Fig. 1, the X-ray source (which is spectrally filtered and collimated to a fan beam), images a slice through an object that is raster scanned through the fan beam. The primary beam (consisting of transmitted X-rays) is recorded on the detector to create a transmission image, whereas the scatter passes through a coded aperture before being recorded by the same detector. The coded aperture imposes a position-dependent modulation on the XRD signal that encodes the location at which the scatter originated^21^. For each acquisition, iterative model-based maximum likelihood estimation is used to recover the XRD spectra at all locations along the fan^20^. As described in Ref.^18^, the system was designed to have a transverse spatial field of view of 15 × 15 cm^2^ with ≈ 1 × 1 mm^2^ spatial resolution and optimized over the momentum transfer range of 0.05–0.3 1/Å with a < 10% fractional momentum transfer resolution. While tomographic imaging is possible with this configuration^20^, we focus here on samples that are both optically thin (less than 3 half-value layers thick^22^) and geometrically thin (inducing a fractional angular scattering uncertainty of less than 5%). This ensures that self-attenuation and multiple scatter are negligible while also maintaining the spatial and momentum transfer resolution goals. For our system, this roughly corresponds to sample thicknesses up to 10 mm.

**Figure 2:**
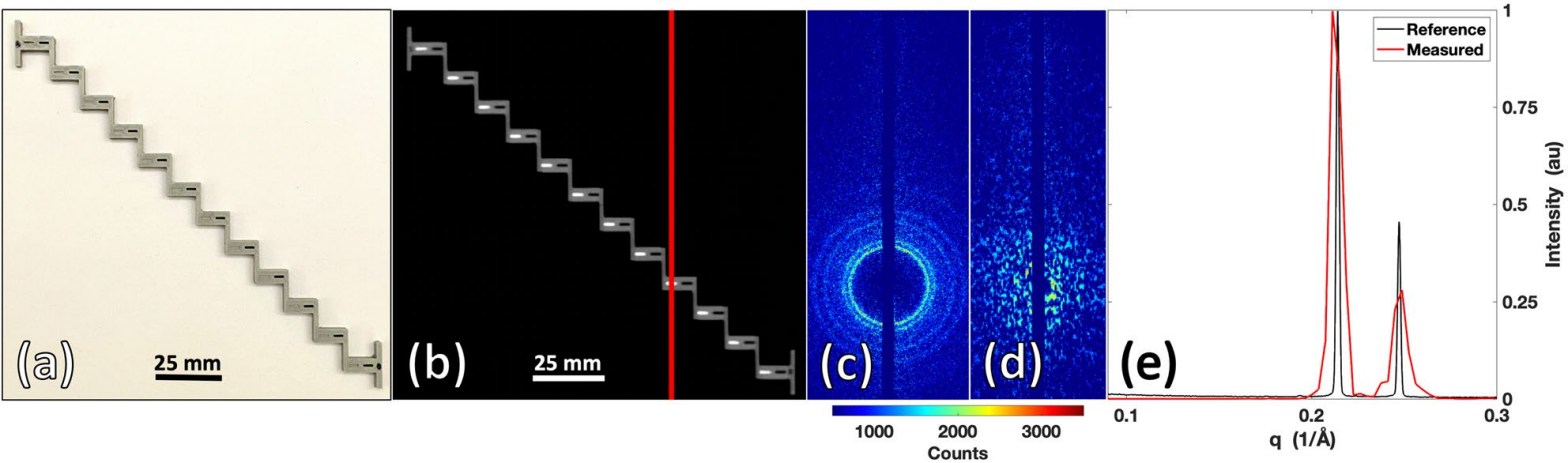
3D printed phantom with 1 × 4 × 3 mm^3^ wells for measuring aluminum powder in the X-ray fan beam coded aperture imaging system. (**a**) Photograph of the phantom. (**b**) X-ray transmission scan of phantom, showing aluminum powder in the left well of each step with right wells empty. (**c**) XRD scatter rings of aluminum (empty PLA well subtracted) without a coded aperture, acquired when fan beam was at the location of the red line in (**b**), showing distinct rings with higher intensity scatter of X-rays. (**d**) Encoded XRD scatter (from aluminum and PLA) passing through a coded aperture, creating spatially unique modulations that can be utilized to reconstruct XRD spectra along the fan beam. (**e**) XRD spectra of aluminum powder measured by inputting the data from (**d**) into a reconstruction algorithm based on the modeled physics of the imaging system, demonstrating accurate identification and measurement of aluminum in the phantom. MATLAB (v2020a) was used for the visual display of all X-ray data presented within this figure and all other figures.

In order to characterize our imaging system’s ability to accurately measure XRD spectral shape and spatial location, a phantom was designed to span the extent of the fan beam with individual wells for solid or liquid materials (see Fig. 2). Polylactic acid (PLA) 3D printer filament was used to create the phantom (Fig. 2a), with two wells designed at each fan location to allow for background subtraction of the scattered signal generated by the PLA plastic. Each well was designed as 1 × 4 × 3 mm^3^ in size, with the left side (signal) wells filled with fine, pure aluminum powder as a reference standard, and the right side (background) wells left empty. A radiograph of the phantom is shown in Fig. 2b, with the vertical red line indicating the slice illuminated in a single, snapshot acquisition. For each acquisition, Fig. 2c,d show the Debye rings produced without and with a coded aperture, respectively. A large fraction of the XRD signal is collected with the area detector, and multiplexed scatter from all objects within the fan beam slice are modulated by the coded aperture and measured in parallel. Figure 2e shows measurements of a single sample acquired using our fan beam coded aperture system (red) and a commercial diffractometer (black), demonstrating excellent agreement between the two measurements. It should be noted that this system was designed to maximize performance in the momentum transfer (q) space ranging from 0.05 to 0.3 Å^−1^, and it is this range that is displayed for all XRD measurements (a measurement with larger q range is shown in supplemental Fig. S2—demonstrating how these measurements are possible although with reduced fidelity).

By repeating this process for all of the wells, we found an XRD transverse spatial resolution capable of resolving the 1 mm of aluminum with a spatial accuracy of ± 0.2 mm over the 15 cm field of view. Similarly, the momentum transfer resolution (q), defined in terms of full-width at half maximum (FWHM) was approximately 0.0095 ± 0.0017 Å^−1^ over a range of 0.05 to 0.3 Å^−1^, with an accuracy of 0.002 ± 0.001 Å^−1^, which agreed well with our prior simulation results^18^. While all of these XRD measurements were made with an acquisition time of 15 s and tube current of 3 mA, we note that higher tube current or lower desired resolutions could decrease this scan time further. For the transmission scan, each fan slice was acquired within 100 ms with a tube current of 6 mA; with the 0.5 mm focal spot limiting the spatial resolution to a few hundred microns (see supplemental Fig. S3). Thus, using this simple but well-characterized calibration phantom, we demonstrated and quantified our fan beam coded aperture imaging system’s resolution and fidelity (for both X-ray transmission and XRD imaging). An additional measurement is shown in supplemental Fig. S4 providing further insight into how spatial resolution can be characterized for this type of imaging system while verifying a spatial resolution of approximately 1 × 1 mm^2^.

**Figure 3:**
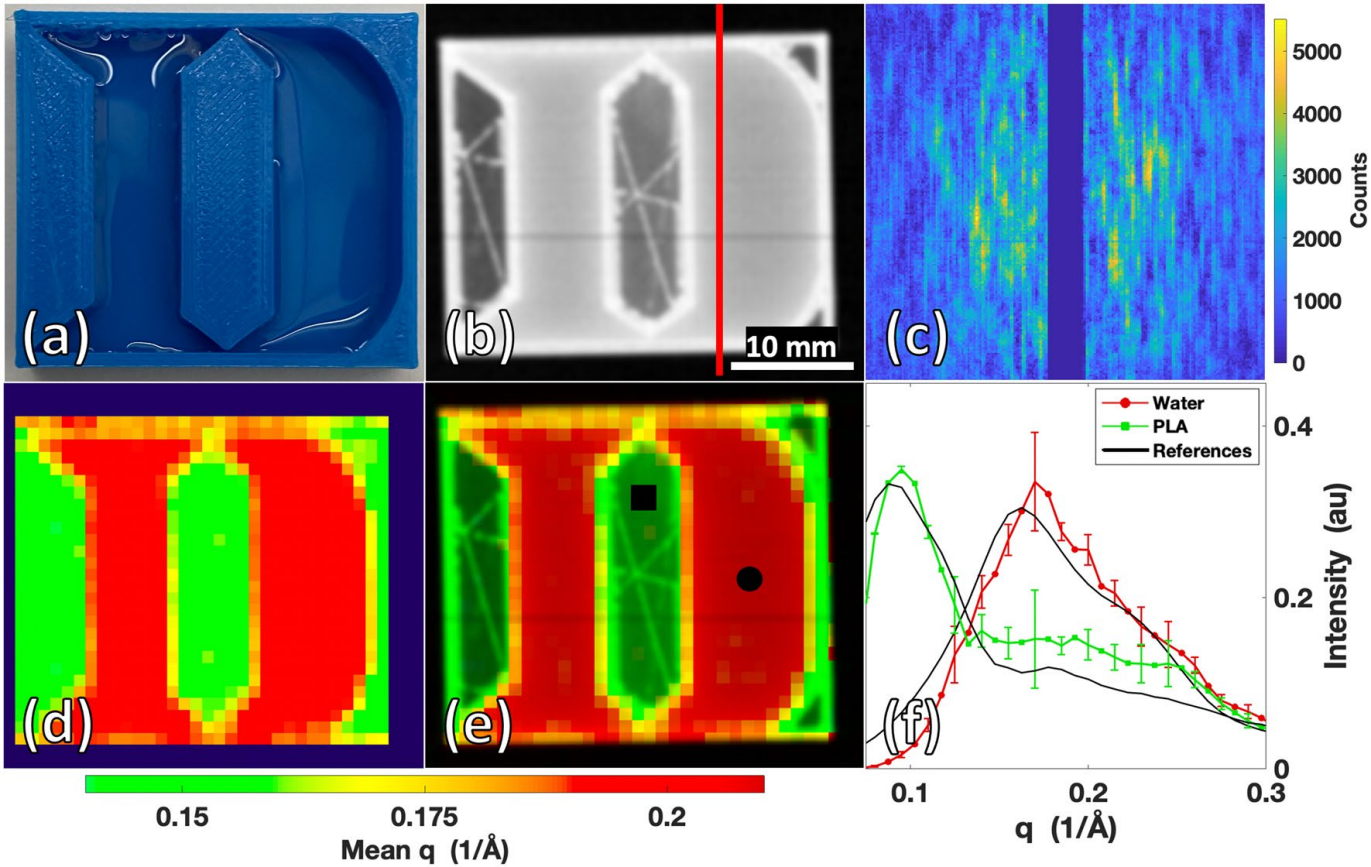
Duke D phantom composed of PLA plastic and water scanned by the X-ray fan beam coded aperture imaging system. (**a**) Photograph of Duke D phantom. (**b**) Transmission scan of Duke D Phantom. (**c**) Multiplexed XRD data acquired when fan beam was at the location of the red line in (**b)**. (**d**) XRD image with artificial coloring based on the mean momentum transfer (q) value of each voxel’s XRD spectrum. (**e**) Combined transmission + momentum transfer (q) colorized (TQC) image of Duke D. (**f**) Mean XRD spectrum obtained from the marked 3 × 3 voxel regions in (**e**) (with standard deviation bars), compared against the XRD spectrum of PLA and water measured in a commercial diffractometer.

Having demonstrated accurate XRD measurement throughout the extent of the fan beam over location and time (by the phantom in Fig. 2 as well as an additional one shown in supplemental Fig. S5), a more spatially complex object was created to test the system’s XRD imaging performance. A 30 × 30 × 6.5 mm^3^ phantom in the shape of the Duke University “D” logo was 3D printed, with the Duke D created as a 5 mm deep well for filling with water (see Fig. 3a). The Duke D phantom was 10% infill, as shown in the radiograph in Fig. 3b by the hollow internal regions with triangulated supports inside the PLA. In the transmission image, the internal structural support of the phantom and overall shape is visible along with the differing X-ray attenuations of the vertical walls of the object vs. the 5 mm thick water or hollow regions. While the phantom contained varying shape and attenuation values, the transmission image alone was unable to uniquely identify or differentiate the materials as PLA and water. Multiplexed XRD measurements were made for each fan slice (as shown in Fig. 3c) to complement the information contained within the radiograph and enable material identification. After reconstruction, an XRD image was created using measured XRD spectra for each object voxel. To view this hyperspectral XRD datacube in a simplified view, despite each pixel containing a continuous spectrum of values, Fig. 3d shows the image in false coloring based on the mean q value of each voxel’s XRD spectrum. By this simple coloration approach, the presence of two unique materials can be identified within the image based on their molecular signatures.

The image shown in Fig. 3e is produced by multiplying the Transmission image with q Colorization (TQC image); this image provides improved material contrast by combining the finer spatial resolution and attenuation information of the transmission image with the molecular structure-based artificial coloring. Comparing a voxel’s measured XRD spectrum against a known ground truth (from literature or commercial diffractometer measurements using a Bruker D2 Phaser system in our lab) allows for quantitative evaluation of an XRD voxel’s material composition. Shown in Fig. 3f is a comparison of the recovered mean XRD spectra measured by the X-ray fan beam coded aperture system with plotted deviation from within the 3 × 3 voxel regions marked in Fig. 3e, compared against commercial diffractometer measurements of the same materials. A cross-correlation of 96.5% for water and 94.2% for PLA was achieved between X-ray fan beam coded aperture and commercial diffractometer measurements, demonstrating high accuracy in material identification from an image that was acquired in only 8 min.

Having demonstrated the X-ray fan beam coded aperture imaging system’s ability to obtain accurate transmission and XRD images for known, controlled phantoms, we next sought to explore the system’s performance in three real-world practical applications including geology, pharmacy, and medicine. Implementation of rapidly acquired X-ray transmission and XRD images in these fields could greatly further scientific exploration and imaging task capabilities.

### Exploration of geological application

We first investigated the system’s ability to characterize geological samples, which is a well-established application of XRD^5,9,10,15,23^. Three rock specimens—fieldstone, asphalt, and limestone—were collected and scanned to evaluate the system’s ability to acquire XRD data from complex, structured samples without any additional sample preparation.

**Figure 4:**
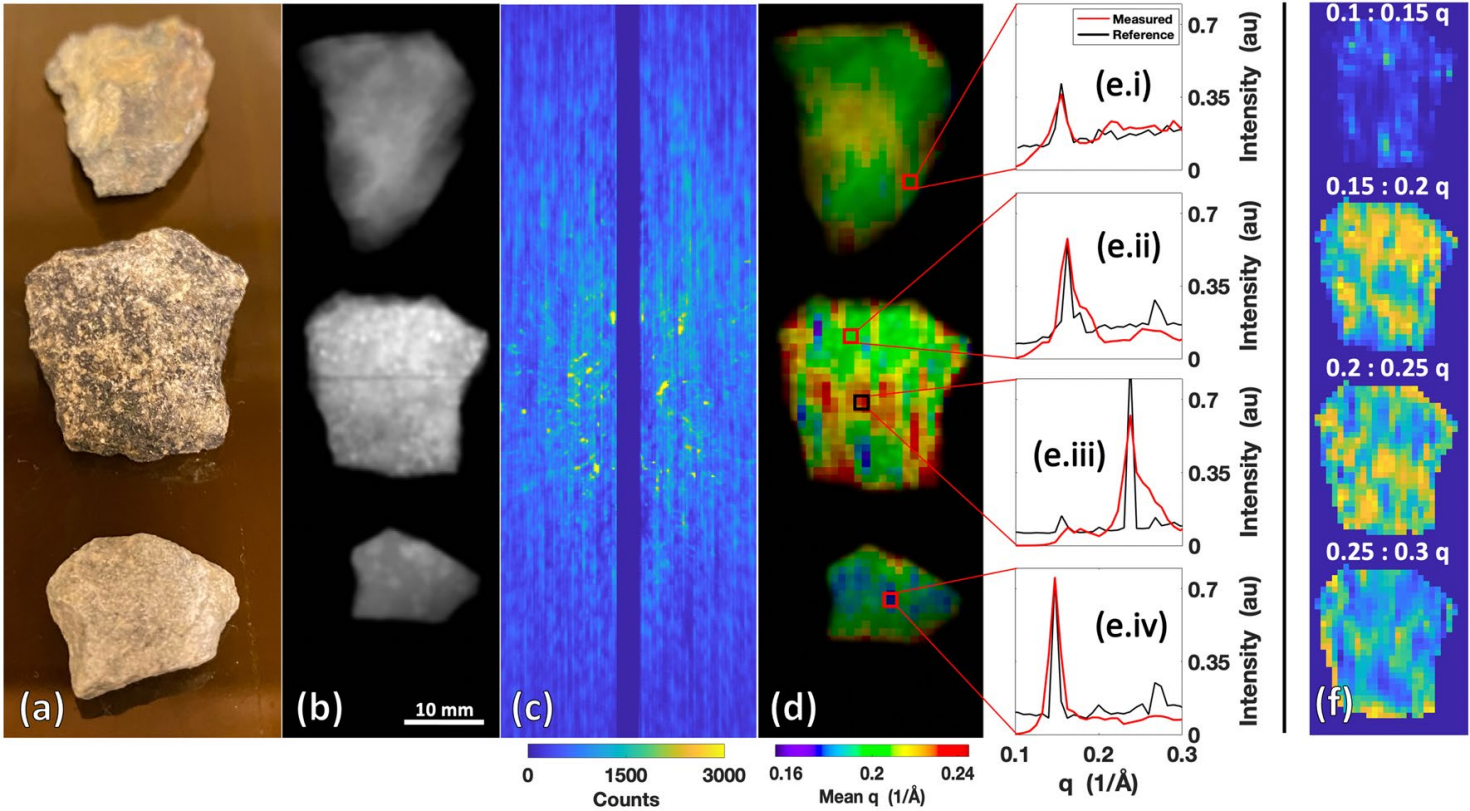
Rock specimens scanned by the X-ray fan beam coded aperture imaging system. (**a**) Photograph of the fieldstone (top), asphalt (middle), and limestone (bottom). (**b**) Transmission scan of the rock specimens. (**c**) Raw multiplexed scatter data from central fan slice showing the textured signal from the rocks. (**d**) Combined transmission + q colorization (TQC) image of the rocks showing new spatial variations relative to transmission alone. (**e**.**i**–**iv**) XRD spectra extracted from the marked locations in (**d**), compared against commercial diffractometer measurements of the rock specimens. (**f**) Hyperspectral display of the asphalt rock (selected due to its higher XRD spatial variation) over distinct q windows.

Shown in Fig. 4a are specimens of fieldstone (top), asphalt (middle), and limestone (bottom) that were scanned using our imaging system. The specimens ranged between 5–10 mm in thickness. Figure 4b shows the transmission image highlighting the variability in attenuation between the different rock specimens. Figure 4c shows the raw multiplexed XRD scatter data obtained from the central fan slice of the rocks. The center of the image in Fig. 4c shows bright peaks in regions where textured/nonconcentric scatter signal from the rocks (especially asphalt) causes bright peaks without radial symmetry. This type of texturing, due to the orientation-dependent crystallinity of the sample, is typically not preserved in commercial diffractometers, where the samples are turned into fine powders prior for XRD scanning. Our method, however, does not require this type of sample preparation and, as such, preserves these texturing effects and facilitates their visualization.

The TQC image in Fig. 4d shows spatial variability of the measured XRD spectra within individual rocks that arises due to variability in the samples’ composition and/or texturing from crystalline structure/orientation. Measurements of chips from each rock at specific locations (marked in Fig. 4d) were made using a commercial diffractometer and plotted in Fig. 4e.i–iv for comparison against our fan beam coded aperture scans. Cross-correlation of the X-ray fan beam coded aperture scan (which required 8 min) and commercial diffractometer measurements (which required 6 min per spot) were 94.4%, 90.4%, 81.3%, and 86.9% for the four spots measured. While we see strong agreement between our measurements and the diffractometer measurements, we do not expect a perfect match between the two. One source of variability between the X-ray fan beam coded aperture vs. commercial diffractometer measurements for these rocks is that the commercial diffractometer only collects XRD data at the surface of the rock, whereas the X-ray fan beam coded aperture measurement averages the spectra throughout the 1 × 1 × 5 mm^3^ voxel. In addition, the sensitivity of the relative peak amplitudes to crystalline orientation for textured samples^24^ presents additional, anticipated deviations from the reference. Despite the textured nature of the rocks’ XRD data and the higher X-ray attenuation relative to the phantom data, our system still produced XRD measurements that agreed with the commercial diffractometer measurements and literature (see supplemental Fig. S6 for additional examples of crystalline material measurements).

For the asphalt rock, Fig. 4f shows hyperspectral images of the obtained XRD signal for different q ranges. The asphalt’s higher level of mean q variation in Fig. 4d (relative to other rocks) motivated utilizing this display to allow for further visualization of the asphalt XRD spectral variability. This hyperspectral image allows for intensity throughout the full XRD spectra to be displayed, thus providing a more complete spatio-spectral XRD map of the specimen. Combination of XRD images with transmission images can provide data beyond that which is provided by transmission alone, as demonstrated in Fig. 4d,f illuminating unique spatial structures. Future applications of this technology on geological samples could use each pixel’s XRD spectrum to assess the material’s composition and quality, without sample destruction.

### Exploration of pharmaceutical application

**Figure 5:**
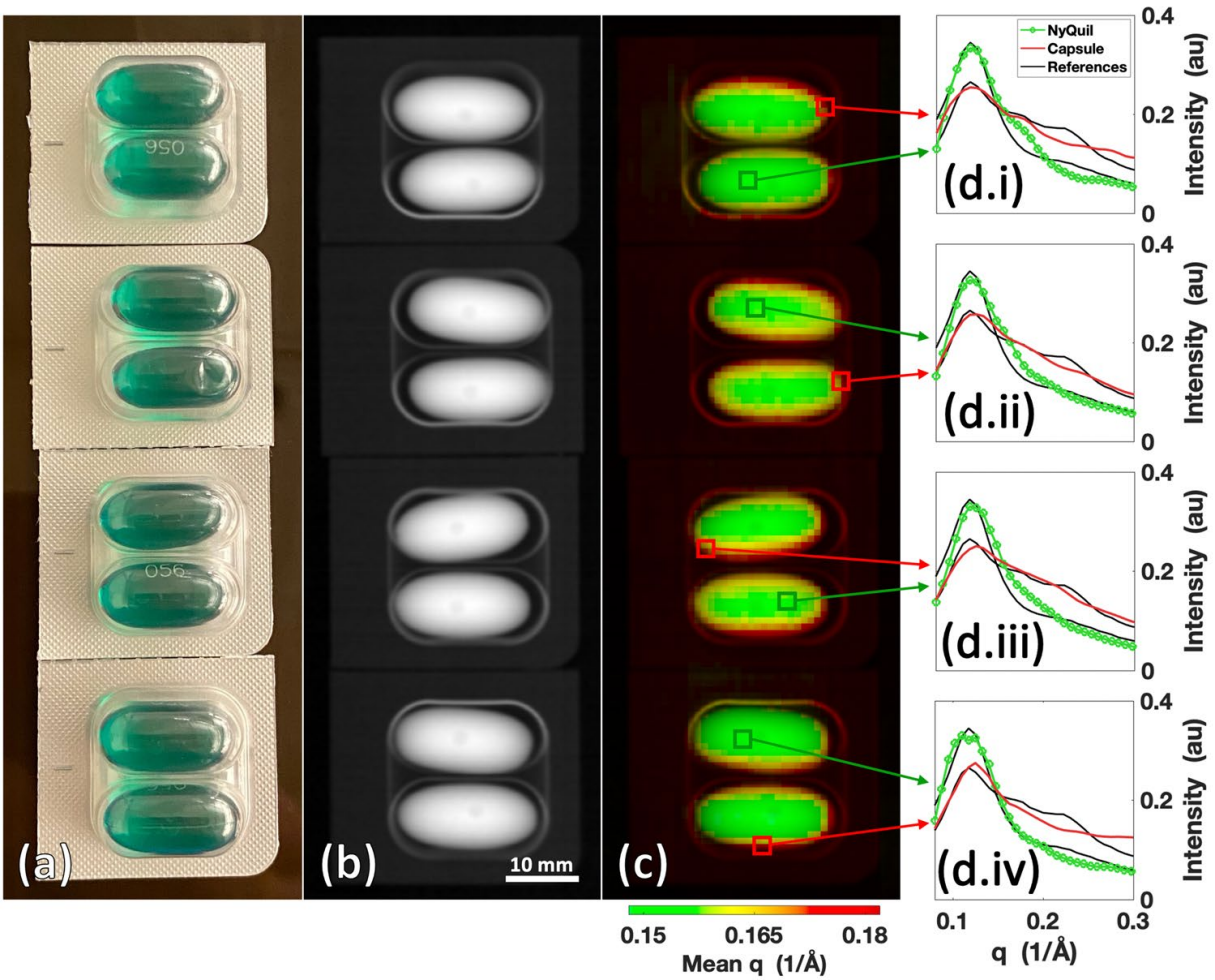
Packaged NyQuil pills scanned by the X-ray fan beam coded aperture imaging system. (**a**) Photograph of the packaged NyQuil pills. (**b**) Transmission scan of packaged NyQuil pills. (**c**) Combined transmission + mean q colorization (TQC) image of NyQuil pills. (**d.i**–**iv**) XRD spectra from each package for individual voxels predominantly containing liquid NyQuil and soft gel capsule compared against commercial diffractometer measurements of each material.

Another application of our system is in the analysis and detection of pharmaceutical drugs (especially when they are contained within packaging or otherwise concealed)^6–8,25–27^. Standard diffractometers are limited to surface-level measurements, which restricts the ability to analyze the spatial variability in composition and/or quality of the drug. In cases where pills are coated with another substance or are inside a container, an inability to collect data directly from the active drug can cause a commercial diffractometer to misclassify a pill. To demonstrate our system’s ability to overcome these limitations, liquid NyQuil pills sealed in their original packaging (≈ 10 mm thick) were scanned using our imaging system to demonstrate an ability to measure XRD spectra of an active drug through capsulation and packaging. The results of the scan are shown in Fig. 5.

Figure 5a shows a photograph of the packaged pills. Within the transmission image in Fig. 5b, the pills vs. their packaging could be identified and differentiated based on density, although the density/shape information did not make it readily apparent that the pills were a liquid drug contained within a soft gel capsule. On the other hand, due to the higher average scattering angle of the capsule compared to the drug, the TQC image in Fig. 5c showed sharp contrast between the capsule and the liquid drug content. Due to the ≈ 1 mm XRD spatial resolution of the imaging system, partial volume effects (in which the XRD spectra of the two materials are mixed) could be observed at the boundaries of the capsules and active drug where the XRD spectra of the two materials were mixed. Comparing the X-ray fan beam coded aperture measured XRD spectra of the packaged capsule and liquid drug against commercial diffractometer measurements of opened and separated capsule/drug (see Fig. 5d.i–iv), average cross-correlation of 99.3 ± 0.2% (NyQuil) and 99.6 ± 0.2% (capsule) was achieved from a 9-min scan. Measurement techniques that identify materials based on surface-level data would require the process and time of opening the packaging and removing the capsules/coating of pills to collect data from the active drug, while the X-ray fan beam coded aperture imaging system can achieve measurement of active drug XRD spectra without these steps or destruction of the sample.

### Exploration of medical application

**Figure 6:**
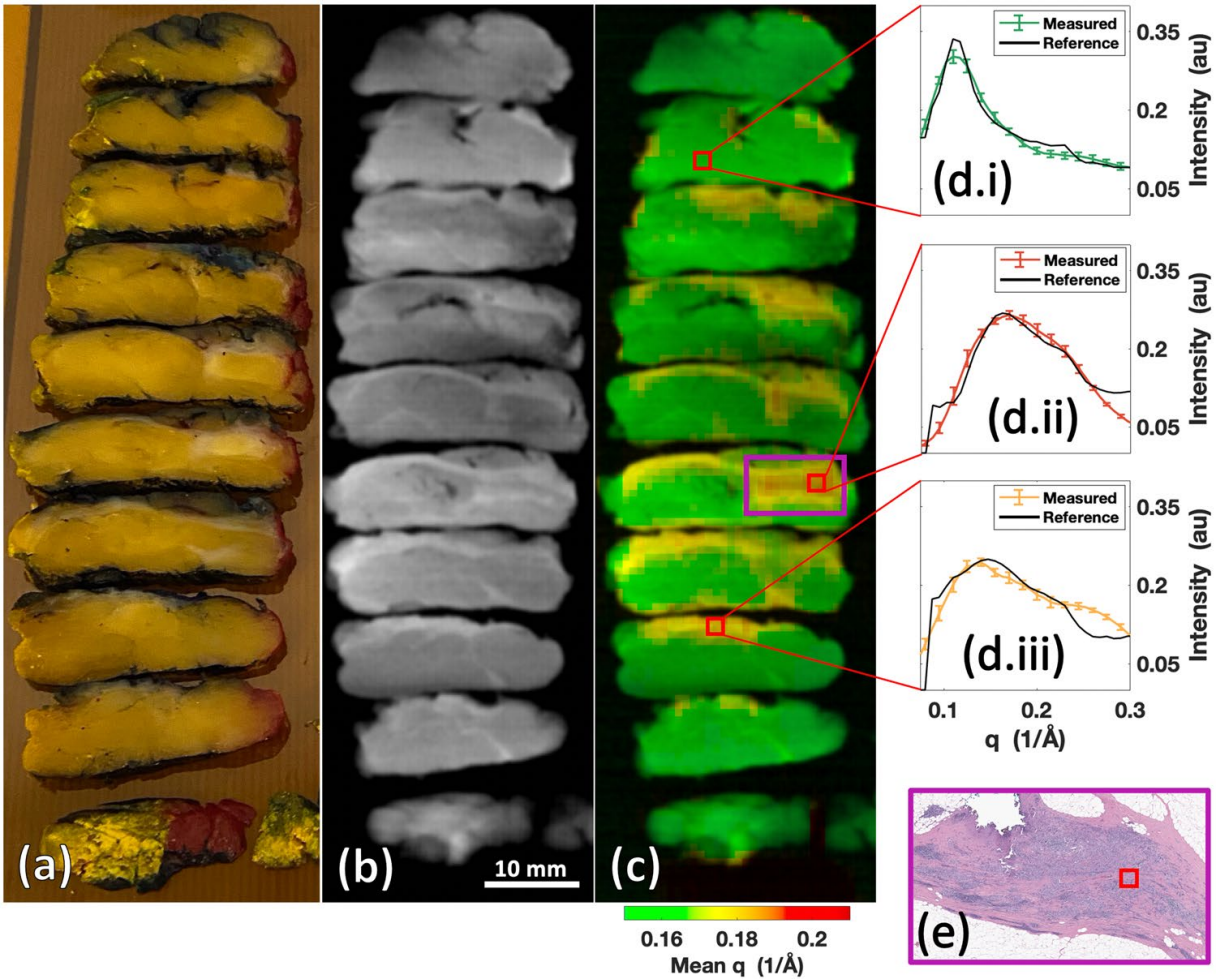
Malignant breast lumpectomy scanned by the X-ray fan beam coded aperture imaging system. (**a**) Photograph of the formalin-fixed and 5 mm thick sliced breast tissue. (**b**) Transmission scan of the breast tissue. (**c**) Combined transmission + mean q colorization (TQC) image of the breast tissue, with mean momentum transfer (q) color highlighting different tissue regions. (**d**) XRD spectra corresponding to voxels at marked locations in (**c**), with references (black lines) derived from literature^28^ of (**d.i**) 65% adipose + 35% fibroglandular, (**d.ii**) 100% cancer, and (**d.iii**) 100% fibroglandular. (**e**) Pathology H&E slide for region of purple box in (**c**), where pathologist confirmed cancerous cells are present. The red square notes where the XRD spectrum was pulled for display in (**d.ii**).

Conventional XRD measurements have previously been utilized for analysis of biospecimens and tissue classification^12,28–32^. Specifically, XRD has been used to study calcifications, bones, and kidney stones, while also demonstrating an ability to differentiate soft tissues including adipose, fibroglandular, and cancer with higher sensitivity and specificity relative to X-ray transmission data. However, implementing XRD imaging in a clinical workflow has faced challenges, including the size of the machine (it must fit in a clinic or laboratory space), scan time (on the order of minutes), and the achievable XRD spatial resolution (millimeter scale or better). As a final demonstration of the performance of our X-ray fan beam coded aperture imaging system, a breast cancer lumpectomy specimen was scanned to demonstrate the system’s ability to identify cancer for assisting pathologists and surgeons in assessing surgical cancer margins. Duke University Surgical Pathology provided a formalin fixed breast lumpectomy specimen cut into 5 mm thick slices (see Fig. 6a).

The transmission image in Fig. 6b shows some spatial structure and varying densities, indicating the presence of different tissue types within the lumpectomy specimen. However, the identities of the tissues were not readily apparent because X-ray attenuation/density are not sensitive indicators of tissue types^33^. By adding the XRD data for mean q coloration in Fig. 6c, the increased contrast shows the presence of 3 different tissues. By extracting the XRD spectra from these differently colored regions and comparing against reported breast tissue XRD spectra in literature^28^ (see Fig. 6d.i–iii), specific tissue compositions were identified. The measured spectra shown in Fig. 6d.i–iii were extracted from 3 × 3 XRD voxel regions (3 × 3 × 5 mm^3^ of tissue total) from local regions believed to predominantly contain a single tissue type to provide a visualization of mean breast tissue XRD spectra. The mean XRD spectrum in Fig. 6d.i (corresponding to the green region) was 98.9% cross-correlated to a weighted mixture of 65% adipose, 35% fibroglandular tissue (close to the common definition of “normal” breast tissue being a 50:50 mix of adipose and fibroglandular). In Fig. 6d.ii, the fan beam coded aperture measured mean XRD spectrum (corresponding to the red region) was 98.3% cross-correlated with the literature XRD spectrum for cancerous breast tissue. Figure 6d.iii shows a 98.8% cross-correlation agreement between our mean XRD spectrum (corresponding to the yellow region) and literature XRD spectrum for fibroglandular breast tissue.

These findings demonstrate our X-ray fan beam coded aperture imaging system’s ability to measure human tissue XRD spectra that match those previously reported in literature. To validate the efficacy of identifying tissue types by matching measured XRD spectra with known literature measurements, we obtained pathologist assessment of an H&E slide (Fig. 6e), which confirmed that the red mass region contains cancer. By providing a method to rapidly differentiate soft tissue/precisely identify cancer among benign tissues in X-ray scans (scan occurred in 7-min), the X-ray fan beam coded aperture imaging system could be used for analyzing biospecimens for pathological, surgical, or radiological applications.

## Discussion

This work demonstrates a fast, high-resolution multimodal X-ray transmission and diffraction imaging technology applied currently to thin samples (up to 10 mm thick) with a wide variety of material compositions while requiring minimal sample preparation. The X-ray fan beam coded aperture imaging system combines high-resolution transmission imaging with coded aperture X-ray diffraction using predominantly off-the shelf components in a compact, user-friendly design that is appropriate for industrial, laboratory, and clinical applications. Consistent with expectations from previous design analyses, the constructed X-ray fan beam coded aperture imaging system demonstrated a ≈ 1 × 1 mm^2^ XRD spatial resolution with > 95% XRD spectral accuracy in scans operating at 15 s/150 mm fan slice acquisition speeds. The full 15 × 15 cm^2^ field of view can be scanned in approximately 30 min, with smaller fields of view such as those shown in Figs. 3, 4, 5, and 6 requiring proportionally less time (under 10 min). For materials including plastics, water, aluminum, crystalline/textured rocks, packaged drugs, and human tissue specimens, we demonstrated that the combination of X-ray transmission data with fast XRD images provides the capability of producing hyperspectral X-ray images that can be used to precisely characterize/identify materials spatially throughout a sample.

The design of the system discussed here, including the choice of component locations, fan beam extent, peak X-ray spectrum energy, filtration, and coded aperture pattern, was based on simulation studies targeting the resolution and field of view reported above. However, future systems can be optimized for different tasks. For example, a system with a longer fan beam extent could be constructed to allow for imaging of larger objects/increased throughput, while smaller coded aperture features and different geometries/magnifications could allow for finer resolutions. Additional trade-off spaces include increased spectral beam filtration for better momentum transfer resolution at the cost of increased scan times. One could also adjust the peak X-ray energy (determined by selected X-ray source/filtration) and component placement in order to vary the penetration depth while optimizing SNR. Finally, we note that a key limiting factor in speed is the current detector’s noise; while an energy-integrating detector was used within our system, photon-counting or energy-sensitive detectors could allow for higher SNR, resulting in significantly faster scan times and improved resolution at the cost of more expensive components (such as large 2D energy-sensitive detector arrays^34,35^). Further simulations combined with the existing experimental system can be performed to optimize the system performance to a particular application and set of constraints.

Another advantage of the system presented here is the ability to rapidly acquire co-registered X-ray transmission and diffraction images. This approach allows one to take advantage of the numerous advances in automatic medical image analysis/cancer detection due to machine learning methods applied on standard transmission images^36–38^ and XRD data^39–41^ while also applying conventional hyperspectral imaging tools for performing material characterization and identification^42–44^. Thus, while we have demonstrated in this paper the imaging capability of our system, the wealth of data provided in the recovered images combining transmission and XRD measurements could provide a significant boost to the performance of these applications by simultaneously providing density information and unique molecular structure-based information for analysis using machine learning models. These approaches, especially those focused on spectral unmixing, have the potential to overcome the system’s XRD spatial resolution and aid in detecting materials that are smaller than the planar or axial resolution. Future work will explore reconstruction and classification algorithms that utilize both transmission and XRD datasets for improved performance.

While all the XRD images in this paper displayed reconstructed XRD data in a planar manner for thin objects (≤ 10 mm thick), the coded aperture additionally provides the ability of performing depth-resolved imaging^20^, which we are currently exploring for this system. Future work will involve thicker objects and explore the imaging system’s volumetric imaging performance. As the thickness of the scanned materials increases, the spatial and q resolutions may be impacted. To maintain imaging quality for thicker samples it will also be important to account for non-uniform self-attenuation along with multiple scatter (which becomes a prominent effect for objects > 10 cm water-equivalent thicknesses^22^). This hyperspectral 3D X-ray imaging capability has the potential to open new research avenues and applications (while minimizing/eliminating sample preparation) including anthropological analysis of fossils/ancient artifacts^45–47^, concealed drug detection within thick packages^26,48^, and fast cancer margin assessment^13,49^ of entire excised tissues without slicing. As we have shown, fast, high-resolution multimodal X-ray transmission and diffraction imaging offers improvements to a multitude of scientific, industrial and medical applications.

## Methods

### X-ray fan beam coded aperture imaging system key components and operation parameters

The overall system fits in a frame of dimensions 51.5 × 22.5 × 28.25 inches (HxDxW). A Spellman XRB x4111 X-ray generator (tungsten anode) was used as the X-ray source for the imaging system, with the X-ray beam facing upwards. This generator has a 0.5 mm focal spot that is capable of operating at 480 W continually, with selectable tube voltages and currents ranging from 30–160 kVp and 0.3–6 mA, respectively. The output was shaped using a multi-stage collimator (to create a 150 × 1 mm^2^ fan beam at the object scan plane) and a 50 µm foil of hafnium placed on the generator exit window (to generate a spectrum peaked at 60 keV with an effective width of 0.5 keV). During transmission data acquisition, the system was operated at 80 kVp, 6 mA, and 100 ms exposures; during XRD data acquisition, the system was operated at 160 kVp, 3 mA, and 15 s exposures (with a lead beam block placed in the primary beam to prevent detector saturation from the primary beam). Internal filtration of the X-ray source included 0.75 mm of beryllium and 1 mm of aluminum, leading to peak detected scattered photon counts of ≈ 2,500 per pixel for most scanned objects when operated in XRD data acquisition mode. For both transmission and XRD data acquisition, the system was operated in a step-and-shoot manner, acquiring data for a single fan width of a stationary object before translating the object to acquire the next fan slice’s data. Open air scans were taken at the start and end of both transmission and XRD measurements for normalization/background subtraction purposes.

An X-ray detector (Xineos-2329, Teledyne Dalsa), with an active area of 228 × 292 mm^2^ was placed at the top of the system. The pixel pitch of the detector was 50 µm, but this was binned to 100 µm during acquisitions of transmission and scatter data to improve SNR.

A coded aperture was placed between the sample and detectors. The coded aperture was composed of 3 mm thick copper (which provided 100:1 modulation contrast for 60 keV X-rays) and consisted of a 2D random pattern with a 40% open fraction and 0.75 mm square features (which created collimation of 14° angular rejection). Lastly, an 8 mm wide slit was placed in the center of the coded aperture, allowing the primary beam to be measured at the detector without attenuation (see supplemental Fig. S1 for enlargement of a sub-region of the coded aperture shown in Fig. 1).

### X-ray fan beam coded aperture image generation

Data was acquired in the following way: the transmission measurement was made first, followed by the scatter measurement. Brief air scans were made before and after both measurements in order to properly normalize the signal. A previously developed forward model reconstruction algorithm based on a maximum likelihood estimator with a Poisson noise model^19,20,50^ was used to estimate the XRD spectra at each voxel along the fan beam, with the resulting fans of estimated XRD spectra being stitched together sequentially to produce the planar XRD image.

In order to represent the hyperspectral XRD data in a 2D image, the mean momentum transfer value (q) was identified as a variable that could add visual separability of materials. The mean q was computed for the data of each XRD voxel from the range of 0.1 to 0.3 Å^−1^ (a range that showed optimal separability of the materials scanned). Each mean q value was then mapped to a visual colormap (from Ref.^51^), providing visual separability beyond the greyscale intensity used in transmission images. It was noted that while the XRD spatial resolution of the system was around 1 × 1 mm^2^, the transmission resolution was a few hundred microns (limited by the 0.5 mm focal spot as seen in supplemental Fig. S3) while also containing intensity information related to material X-ray attenuation. The mean q images were inherently co-registered to the transmission data, with a simple projection required to map the transmission + mean q colorizing data together for TQC image generation. A multiplication of each pixel’s transmission intensity with the corresponding color data from the mean q image was used to produce the TQC images, providing the benefits of finer spatial resolution, X-ray attenuation information, and molecular-based color information in a single 2D image.

For reference XRD spectra that were not found in literature but instead measured by our group, a Bruker D2 Phaser commercial diffractometer was used for data acquisition. Imaging of the human breast lumpectomy sample (before returning to Duke Medical Center Surgical Pathology) was conducted in accordance with all relevant guidelines and regulations, under a protocol approved by Duke University’s Institutional Review Board—informed consent was obtained from the patient whose tissue was used in the study.

## Supplementary Information

Supplementary Figures.
