# Supplementary material for: X-ray fan beam coded aperture transmission and diffraction imaging for fast material analysis
Source: Sci Rep. 2021 May 19;11:10585. doi: 10.1038/s41598-021-90163-0 (PMC8134570; doi:10.1038/s41598-021-90163-0)
Supplement: Supplementary file 1 — Supplementary Figures. [file 41598_2021_90163_MOESM1_ESM.pdf]

Supplemental Materials

X-ray Fan Beam Coded Aperture Transmission and Diffraction Imaging for Fast Material Analysis

Stefan Stryker [1], Joel A. Greenberg[1,2], Shannon J. McCall[3], and Anuj J. Kapadia[1,4*]

[1]Medical Physics Graduate Program, Duke University;

[2]Department of Electrical and Computer Engineering, Duke University;

[3]Department of Pathology, Duke University Medical Center;

[4]Carl E. Ravin Advanced Imaging Laboratories, Dept. of Radiology, Duke University, Durham, USA, 27708

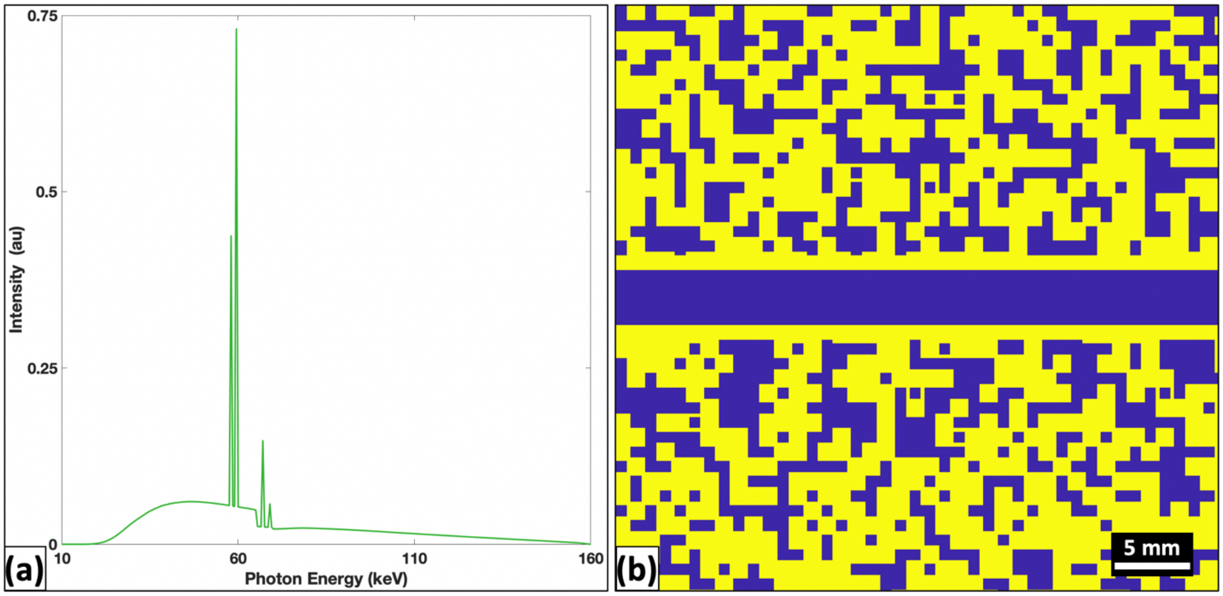

Fig S1 Modeled X-ray source spectrum from tungsten anode with filtration and enlargement of local region in coded aperture. **a)** XSPECT was used to model the X-ray energy distribution for a tungsten anode operated at 160 kVp with intrinsic beam filtration of 1 mm aluminum and 0.75 mm beryllium. Additional filtration consisting of 0.5 mm hafnium was used to further increase the 60 keV peak prominence. The narrow-band spectrum around 60 keV is sufficient to enable angle dispersive XRD measurement with < 10% fractional momentum transfer resolution due to the model-based reconstruction algorithm used. **b)** A zoomed-in look at a sub-region of the coded aperture shown in **Fig 1** to demonstrate the 2D random pattern of copper material (yellow) and open air (blue) for encoding the scatter signal. The central slit allows the primary fan beam to pass to the detector unattenuated for transmission measurements, while the coded aperture on both sides adds spatially dependent information to enable the reconstruction algorithm to localize scatter origin along the fan beam extent.

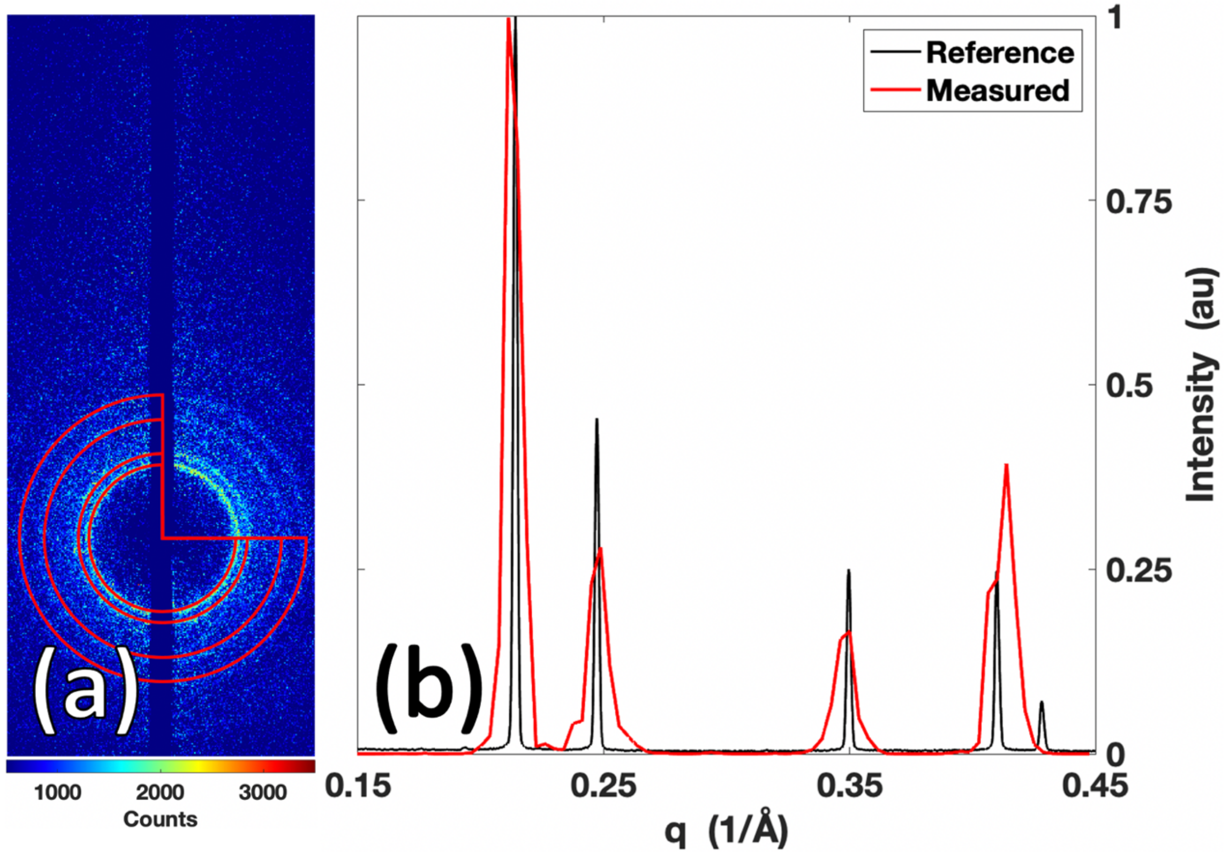

Fig S2 Aluminum powder data from **Fig** 2 with X-ray diffraction (XRD) data shown beyond the system's optimized range of 0.1 to 0.3 momentum transfer (q in 1/Å). **a)** Debye diffraction rings from aluminum powder 1 x 1 x 1 mm^3 'point source', with the first 4 peaks marked by 270-degree arc-length circles for visual identification of the peaks (this is a zoomed-in version of figure 2 c) **b)** Fan beam coded aperture measured XRD spectrum of the aluminum point source recovered from the data in figure 2d (with the coded aperture installed); however, in contrast to figure 2e, the q range is shown out to 0.45 1/Å. While this expanded range is beyond what the system was designed and optimized towards, reasonable peak locations are identified. A slight shift of the 4th peak is observed due to the presence of the 5th peak and the reduced momentum transfer resolution (which scales linearly with momentum transfer). In addition, lower signal to noise at these higher scatter angles could be contributing to this degradation in performance. In consideration of future systems designed towards tasks seeking to work with higher q ranges, longer exposure times, different X-ray energies, and system geometries could be designed towards maintaining spectral quality in this q range currently not under consideration by this works presented prototype.

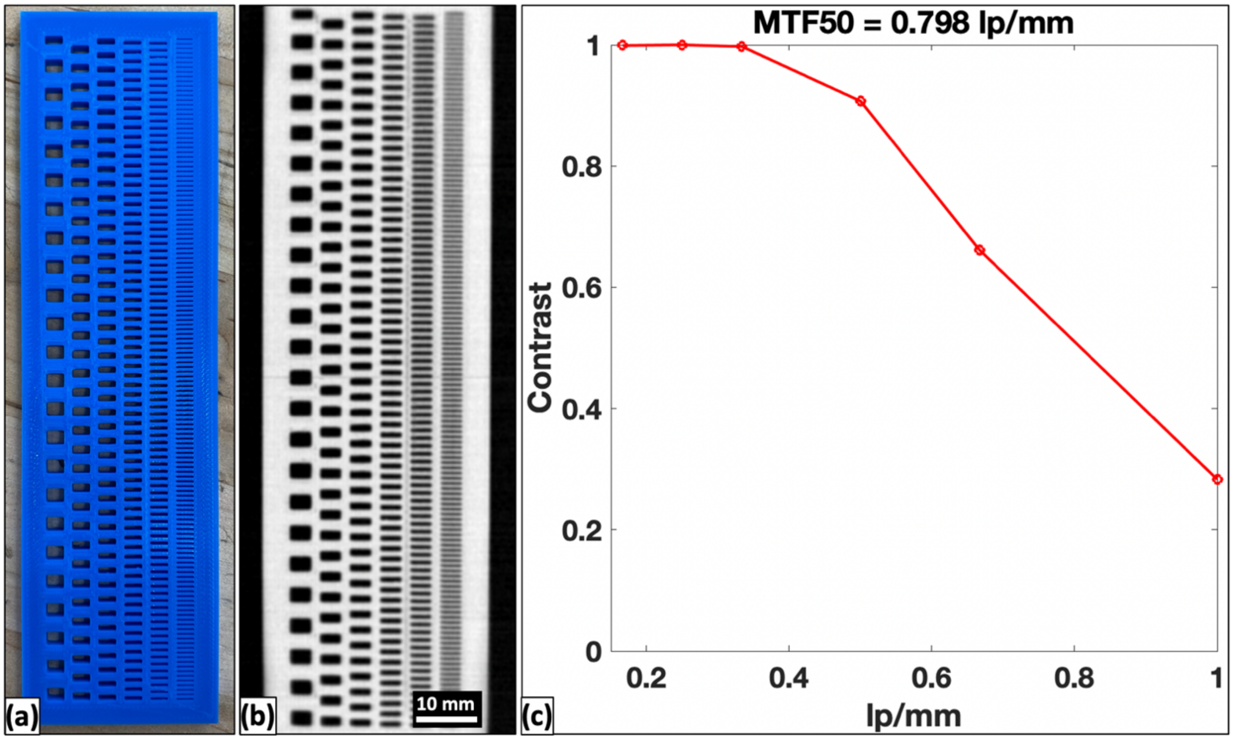

Fig S3 A 3D printed line-pair phantom of PLA plastic and air for measuring the X-ray transmission resolution of the imaging system. **a)** A photograph of the phantom, where bar widths for the columns from left to right are 3, 2, 1.5, 1, 0.75, and 0.5 mm **b)** X-ray transmission scan of the line-pair phantom taken by the X-ray fan beam coded aperture imaging system **c)** An MTF curve generated from the transmission data in **b**, where Michelson contrast was used for the sinusoidal data, and the MTF50 value where contrast has decreased by 50% (often used as a metric for resolution) is 0.798 lp/mm or 0.627 mm, suggesting a transmission spatial resolution slightly above and limited by the 0.5 mm focal spot of the X-ray source.

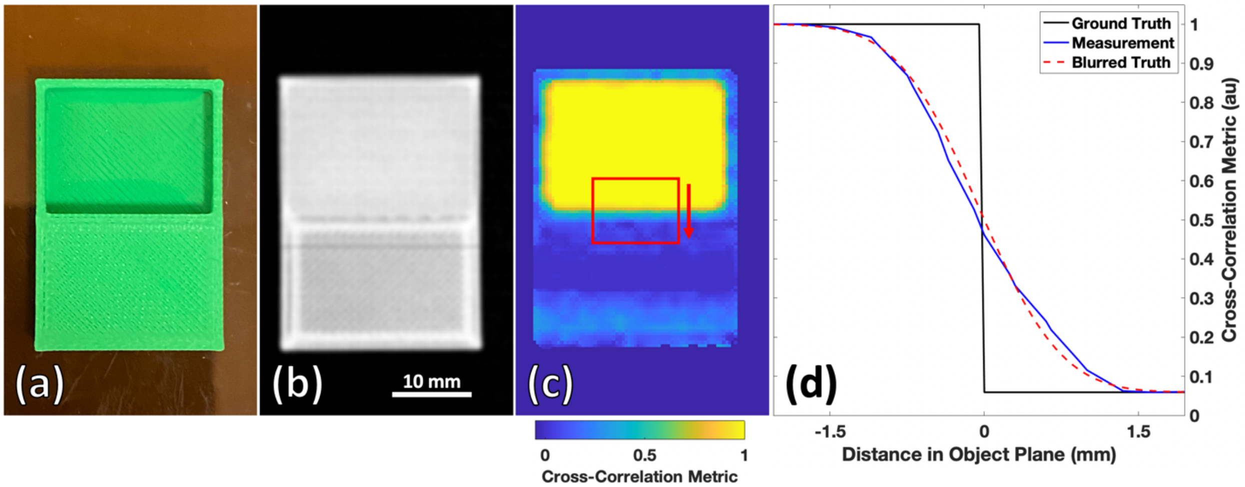

Fig S4 A water and PLA phantom designed for investigating the X-ray diffraction (XRD) spatial resolution of the X-ray fan beam coded aperture imaging system. **a)** Photograph of the phantom with a 15x20x5 mm^3 water well placed above a 15x20x5.5 mm^3 block of PLA **b)** Transmission X-ray scan of the phantom, where attenuation-based contrast does not uniquely identify the presence of water or PLA in the phantom **c)** An image of a cross-correlation based metric (defined in ref[18] from main text) where water was selected as the primary material to identify, creating a normalization where 1 corresponds to measured X-ray diffraction (XRD) spectra matching water while 0 corresponds to matching the XRD spectrum of PLA. The red box region at the boundary of water and PLA shows the region over which columns (i.e., slices along the vertical direction, as indicated) were averaged in order to determine the XRD spatial resolution **d)** Analysis of the data from **c**, where the blue line is the average measurement pulled from the red box region, the black line represents the expected ground truth based on the known boundary location, while the red dashed line represents convolving the ground truth object with a Gaussian curve (full-width-at-half-maximum [FWHM] of 1.4 mm). This Gaussian was found to generate the best match with the measured data, suggesting this prototype's XRD spatial resolution for water and PLA is just above 1 mm. Defining spatial resolution for hyperspectral imaging is a non-trivial task, as grayscale contrast is not along sufficient; rather, one requires a method for analyzing both the intensity and spectral quality at adjacent voxels in the object. As this can depend on a particular task, spectral shape (e.g. material composition), and spatial configuration, there is no 'one-size-fits-all' definition. While any work focused on specific applications should seek to quantify spatial resolution as it relates to the materials/objects that will be present in the use case, we find that our approach here is consistent with the impulse response analysis shown in figure 2. Lastly, we note that this spatial resolution for water-PLA is slightly above the anticipated simulated resolution of 1.1-1.2 mm in ref[18] for comparable materials of adipose and cancer (similar XRD spectra to PLA and water), and this is anticipated due to the 0.5 mm focal spot of the X-ray source (compared to simulation which assumed no focal spot width).

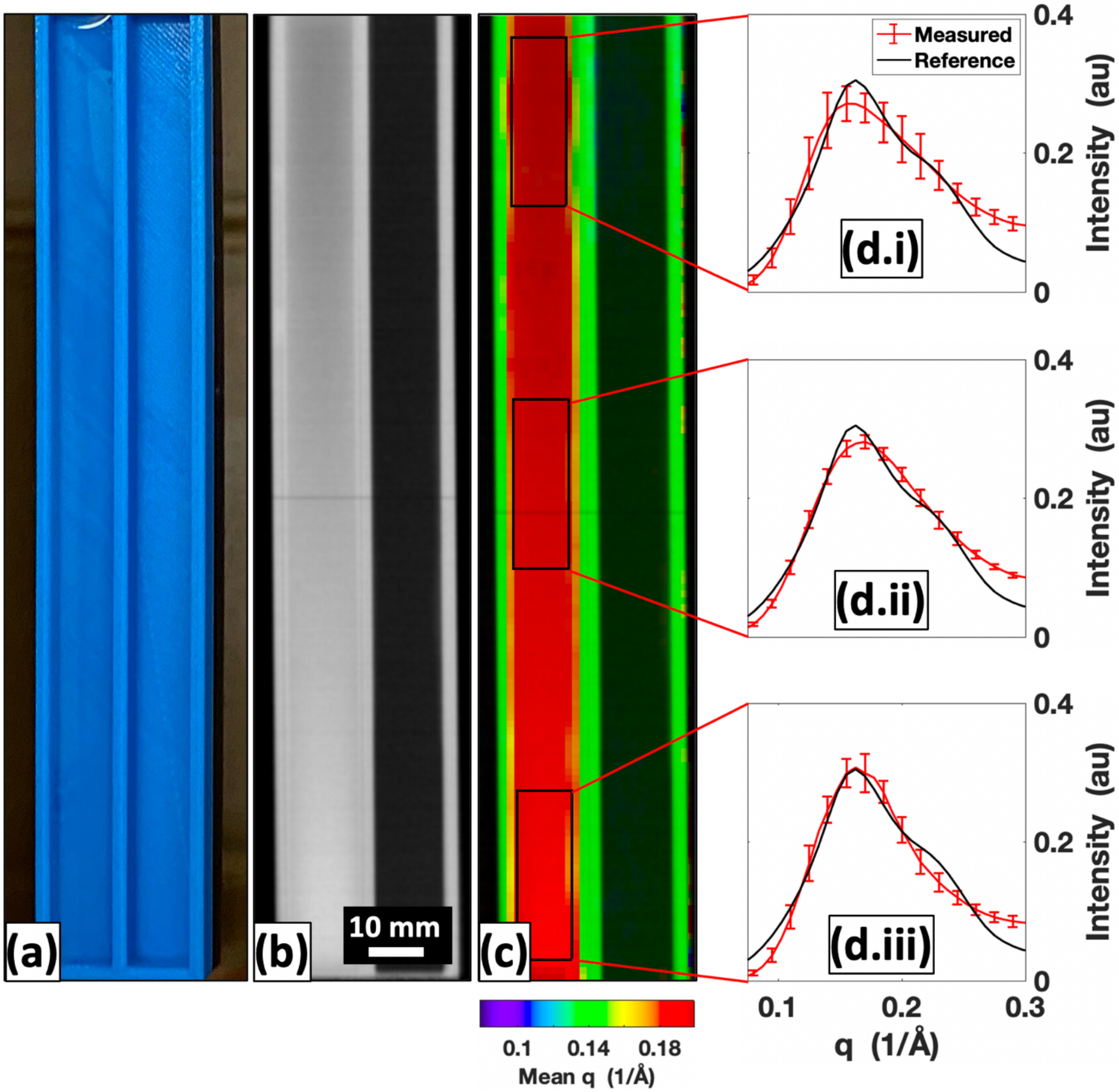

Fig S5 A 3D printed phantom (30 x 155x6 mm^3) with left trough filled with water for validating the consistency of X-ray fan beam coded aperture measured XRD spectra along fan beam extent and over time. **a)** A photograph of the water trough phantom, with water in the left well **b)** X-ray transmission scan of the trough phantom, where a slight tilt relative to gravity can be seen causing there to be higher attenuation (or more water) on the lower extent of the trough relative to the top **c)** A transmission + mean q (TMQ) image of the trough, showing the higher mean q of water spectra in red and the lower mean q of PLA in green **d.i-d.iii)** Mean XRD spectra with standard deviation bars pulled from the 10x30 mm^2 regions marked in **c**, showing slight variations in distributions of reconstructed XRD spectra (measured over 30 mm extents along fan beam and 2.5 min of data collection) while generally agreeing with water's reference XRD spectra measured in a commercial diffractometer.

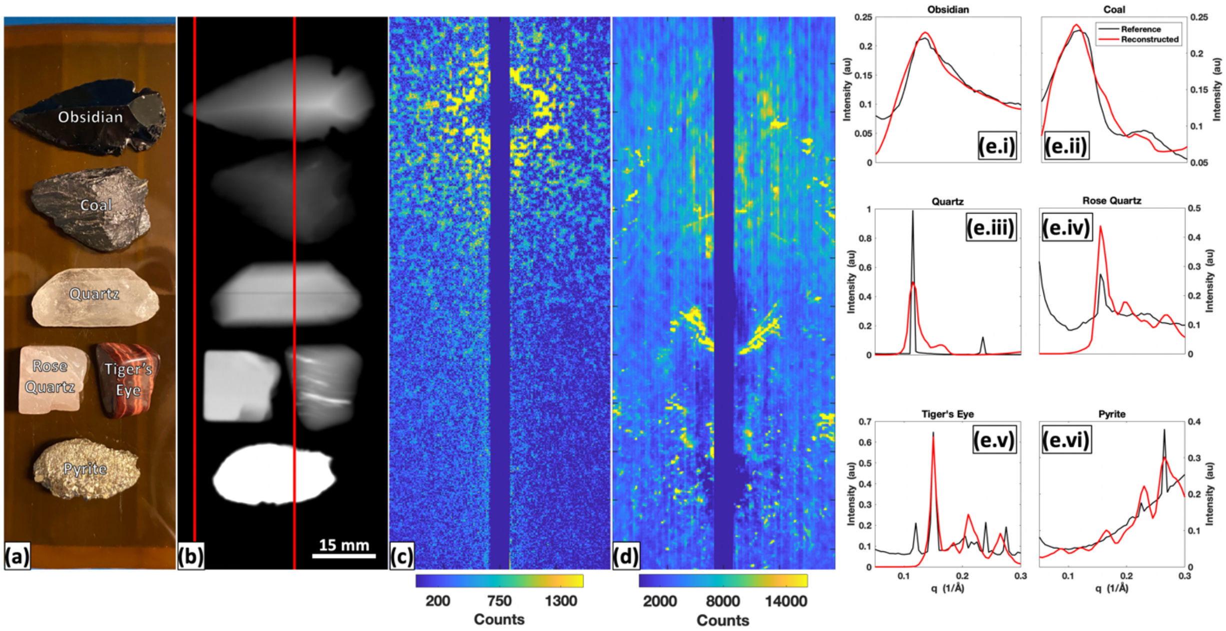

Fig S6 Additional crystalline geological samples imaged by the X-ray fan beam coded aperture imaging system. **a)** Photograph of the rock samples (max thickness of 15 mm, which pushes the boundaries of the system's depth imaging), with labels on each sample identifying them as obsidian, coal, quartz, rose quartz, tiger's eye, and pyrite **b)** Transmission scan of the rock samples, windowed so that most samples could be clearly viewed while the pyrite's highly attenuating nature prevents viewings it clearly in a single window **c)** Raw encoded scatter data from the left red fan slice in **b**, showing the broader scattering of X-rays from obsidian alone **d)** Raw encoded scatter data from the right red fan slice in **b**, showing the textured nature (non-concentric scatter rings) of the data measured from the non-powderized crystalline samples **e.i-vi)** Measured X-ray diffraction (XRD) spectra pulled from individual pixels reconstructed by the X-ray fan beam coded aperture imaging system, along with reference spectra measured in a commercial diffractometer from the surface of the samples at a single spot. Multiple peaks can be seen, with strong agreement with the reference data. However, since the rock specimens have been left in whole form (not powderized, as is common with surface-level commercial diffractometer measurements), it is expected that the fan beam coded aperture XRD imaging measurements (which average the recovered spectra throughout the thickness of the sample) should vary from commercial diffractometer measurements. In addition, orientation-dependent texturing effects will naturally result in different relative peak amplitudes (including missing peaks).
